# Navigating the Complexities of Symptomatic Hydronephrosis in Pregnancy

**DOI:** 10.7759/cureus.61240

**Published:** 2024-05-28

**Authors:** Baidar Khalabazyane, Rotimi David, Rahel Rashid, Joshua Philips

**Affiliations:** 1 Urology, Royal Bournemouth Hospital, Bournemouth, GBR; 2 Urology, Great Western Hospital National Health Service (NHS) Foundation Trust, Swindon, GBR; 3 General and Colorectal Surgery, Arrowe Park Hospital, Wirral, GBR

**Keywords:** ureteric obstruction, pregnancy, ureteric stent, nephrostomy, symptomatic hydronephrosis

## Abstract

The management of symptomatic hydronephrosis presents substantial challenges due to the absence of consensus within clinical guidelines concerning pain management, diagnostic approaches, therapeutic interventions, and follow-up protocols.

This literature review focuses on complexities involving diagnostic challenges that arise from the difficulty in distinguishing physiological from pathological obstruction and treatment complexities that involve deciding on the most appropriate pain management medications and safe interventions while minimizing risks to both the mother and foetus.

To address these challenges, a comprehensive search of electronic databases, including PubMed, Embase, and Google Scholar, was conducted for the terms “hydronephrosis”, “hydronephrosis in pregnancy”, “ionising radiation in pregnancy”, and “safe analgesia in pregnancy”. Moreover, Mendeley software was used to collect and organize the references.

Diagnostic complexities involve selecting the appropriate imaging modality that balances accurate diagnosis with minimal radiation to the foetus. Ultrasound remains the first-line option. However, it has limitations in delineating the underlying aetiology. MRI avoids ionizing radiation but has restricted utility due to foetal movement artifacts. CT provides the highest diagnostic accuracy but raises foetal radiation exposure concerns, though ultra-low dose protocols (<1 mGy) are deemed acceptable by most guidelines.

Management includes either a conservative approach, which is a safe option in the majority of cases, or intervention with a percutaneous nephrostomy or ureteric stent insertion, both with comparable symptom control. However, there is no consensus on the optimal frequency for drain changes to prevent rapid encrustation. Definitive procedures like ureteroscopy and percutaneous nephrolithotomies remain controversial. Most guidelines suggest limiting these interventions to specialist centres during the second trimester if required.

## Introduction and background

Hydronephrosis in pregnancy is a common finding, most of these are asymptomatic and attributed to physiologic or hormonal changes [1]. Physiologically, pregnancy entails an increased cardiac output along with decreased systemic vascular resistance. The renal blood flow rises by 30%, and the glomerular filtration rate doubles [2]. Consequently, asymptomatic hydronephrosis of varying degrees can be found in more than 90% of pregnancies [3].

Maximal dilatation is seen at 24-28 weeks of pregnancy [4]. Hydroureter (and hydronephrosis) is more prominent on the right than the left, and it has been observed in 80% of pregnant women due to the physiological dextrorotation of the gravid uterus and the engorged right ovarian vein draining into the renal vein on the right side [1]. It is also hypothesized that the left ureter is protected from dilation by the loaded sigmoid colon [5]. The dilated collecting system retains an increased amount of 200-300 mL of urine, leading to urinary stasis and a 40% higher risk of developing pyelonephritis in pregnant women compared to non-pregnant women [1].

Symptomatic cases, however, are less frequent (3%) [6]. Clinicians are often faced with multiple challenges when managing these cases, as the presence of the foetus and coexistent physiologic changes of pregnancy introduce peculiar challenges that evolve with changes in gestational age. Management of symptomatic hydronephrosis in pregnancy is complex, requiring multi-specialty collaboration and careful consultation with patients and their families. The literature on this difficult condition is sparse, leading to limited and non-consensus guideline recommendations. Areas of management complexities in symptomatic hydronephrosis of pregnancy include diagnostic dilemmas associated with the choice of imaging modality, difficult choice of renal drainage procedure when required, uncertainties around the timing of definitive intervention, and problems with pain management due to limited choice of safe analgesia in pregnancy.

## Review

Methodology

We conducted a thorough search of the electronic databases in Embase, PubMed, and Google Scholar. Articles were included with key terms “symptomatic hydronephrosis," “analgesia in pregnancy,” “hydronephrosis in pregnancy,” and “ionizing radiation in pregnancy.” We also manually reviewed the available articles to include further references that meet the eligibility criteria. We also reviewed guidelines from prominent urological associations such as the Urological Association of Asia, the NICE guidelines, the European Association of Urology, and the American Urologic Association. Mendeley software was used to collect and organize the references.

Diagnostic challenges

Diagnostic uncertainty remains a major challenge, as the normal physiological changes of pregnancy may mimic ureteric obstruction [7]. A transabdominal (TA) ± transvaginal (TV) ultrasound scan is generally regarded as the first line for diagnostic evaluation as it confirms the laterality and severity of the hydronephrosis [6-8]. Magnetic resonance imaging (MRI) is frequently recommended as a second line, with computed tomography (CT) scans reserved for very limited scenarios.

On Ultrasound

As mentioned earlier, TA and/or TV US are the first-line options. Ultrasonography is widely regarded as a safe diagnostic option for assessing the extent of hydronephrosis in pregnant individuals. Moreover, it offers the advantages of being cost-effective and providing immediate, real-time results. One factor limiting US evaluation is that it cannot easily assess hydronephrosis that has extended beyond the pelvic brim. In these cases, a distal/low ureteric stone is usually blamed as the culprit [9]. Assessing the ureteric jets via the US can augment its diagnostic efficacy, particularly in instances of uncertainty. These jets typically manifest as the outflow of urine from the ureteric orifice into the bladder and are commonly visualized using color Doppler imaging at the bladder base. Nonetheless, approximately 13% of patients, notably in the third trimester, may not exhibit discernible ureteric jets even in the absence of obstruction [10]. A further use of ultrasound, especially color Doppler ultrasound, is in the assessment of renal resistive index (RI). This is a proxy for pressure within the intrarenal vessels and the renal collecting system. This index typically rises in cases of acute ureteric obstruction. A value exceeding 0.70 is deemed clinically significant [11-13].

In summary, hydronephrosis is generally more likely to be pathologic if left-sided, extending beyond the pelvic brim, or associated with obstructive features such as an elevated renal resistive index and/or reduction in the visualized ureteric jets.

On Magnetic Resonance Imaging (MRI)

MRI scan (1.5 Tesla) without gadolinium contrast has been touted as a second-line imaging modality since it can identify the level of obstruction, and stones could show up as filling defects [7]. Typically, in cases of physiological hydronephrosis during pregnancy, obstruction occurs at the sacral promontory, where the ureter becomes compressed between the psoas muscle and the gravid uterus. Should ureteral dilatation extend beyond this juncture, clinicians should entertain the possibility of pathological obstruction [14].

However, MRI may not necessarily offer additional information over ultrasound in characterizing obstructive pathologies, and feto-maternal movements can also degrade MRI image quality. While MRI does not use ionizing radiation, there have been a wide variety of opinions regarding the safety of MRI, specifically around possible damage to the foetal auditory system and tissue warming, specifically in the first trimester. However, recent publications [15] seem to be in consensus that there is no evidence for the safety concerns [16,17] and that no distinct considerations are required regardless of gestational age, even in the first trimester [14,18].

On Computerized Tomography (CT)

A CT scan still has the highest positive predictive value (close to 96%) compared to MRI (80%) and ultrasound (77%) for the diagnosis of the underlying abnormality in pathologic hydronephrosis during pregnancy [7]. This makes a strong case for the use of CT scans in acute conditions, such as symptomatic hydronephrosis, even in pregnant patients. So, if clinically warranted, a CT scan should not be withheld [18]. The use of oral or intravenous (IV) contrast also attracts some discussion. Undoubtedly, they increase a CT scan’s diagnostic capability. Oral contrast agents are non-absorbable by the GI tract and do not pose any actual or theoretical risk. The possible risk in IV contrast is because the most common agents are iodine-based. Due to their ability to cross the placenta [19], iodine-based agents pose a theoretical risk of teratogenesis and mutagenesis, as well as potential effects on the foetal thyroid gland due to iodine exposure [19,20].

Another issue with CT scans is the potential risk of foetal radiation exposure, which depends on the number and spacing of image sections. Utilizing low-dose scans can mitigate this risk.

The quoted foetal dose for an abdominal CT scan is in the range of 4.8 - 5.8mGy [21]. While radiation doses associated with mutagenesis (500mGy) and teratogenesis (50mGy) are well beyond the CT scan range, carcinogenesis can occur at any radiation dose below 10mGy [22]. The risk of carcinogenesis is, however, believed to be low with reduced radiation doses, and the EAU guidelines suggest foetal radiation exposure <0.5mGy does not require any special justification [7].

In the United Kingdom, the Royal College of Radiologists, Royal College of Radiographers, and Health Protection Agency recommend that a foetal radiation dose below 1mGy has only a 1:10,000 risk of childhood cancer, so imaging involving foetal radiation doses within this limit should not be withheld in pregnancy if clinically indicated [23]. The recommendation in the United States from the American Urologic Association, in alignment with the American College of Obstetrics and Gynecology, is that radiation doses up to 50mGy mGy are acceptable in pregnancy if justifiable following clinical evaluation [24,25].

The main advantage of a CT scan is that its incorporation into the diagnostic pathway of symptomatic hydronephrosis in pregnancy will accurately exclude obstruction and prevent unnecessary invasive interventions and negative ureteroscopy in pregnancy. This is the justification for considering an ultra-low-dose CT KUB (0.5 - 1.0mGy) as part of the evaluation in cases of diagnostic uncertainty.

Treatment complexities

Difficulties are encountered not only in making a diagnosis but also in choosing the most appropriate treatment modality while minimizing risks to the mother and fetus. Depending on the aetiology of the hydronephrosis, different management options are available. One of the uncommon but quite significant causes to be considered are kidney and urinary tract stones. There is evidence to suggest that pregnancy does increase the likelihood of experiencing a first-time symptomatic kidney stone. This risk reaches its peak nearing delivery and gradually diminishes, typically returning to baseline levels within one year postpartum [26].

Conservative management is successful in the vast majority of cases. This entails the use of a combination of fluids, antibiotics, analgesia, and bed rest.

In terms of renal drainage, if indicated, there seems to be a consensus across most guidelines that percutaneous nephrostomy and retrograde ureteric stenting are comparable for symptom control [7,25]. To limit radiation exposure, either option can be performed under ultrasound or pulsed-fluoroscopy guidance. However, the optimal frequency of drainage tube changes is unclear, despite the propensity for rapid encrustation from absorptive hypercalciuria, hyperoxaluria, hyperuricosuria, bacteriuria, and other metabolic changes in pregnancy. The main urological guidelines offer limited direction in this regard, though some authors have suggested regular changes every 4-6 weeks [27]. In our experience, given the lack of clear guidelines, it appears optimal to replace drainage tubes as soon as logistically possible and safely, while titrating the timing of future changes depending on the severity of encrustations encountered.

Another significant issue is whether to attempt definitive ureteric unblocking surgery such as pyeloplasty or ureteroscopy during pregnancy. While there seems to be no evidence to support pyeloplasty in pregnancy, some guidelines recommend ureteroscopy during the second trimester, but only in specialist centres with easy access to ancillary and obstetric services (Table 1). If going ahead with ureteroscopy, it is important to ensure a 15-degree head-up tilt of the patient to reduce aortocaval compression and supine hypotension syndrome. Ureteroscopy can also be carried out under ultrasound guidance to reduce radiation exposure from fluoroscopy. The procedure, however, has an overall complication rate of about 5% in some series, so the potential benefits need to be carefully balanced against the risks involved [28]. Extracorporeal shockwave lithotripsy remains contraindicated in pregnancy, but percutaneous nephrolithotomy (PCNL) is not. There are a few reports of PCNL in the literature, with most of these being done in the supine position with ultrasound-guided kidney puncture [29].﻿

**Table 1:** Meta-synthesis of major urology guideline recommendations on management of obstructive hydronephrosis in pregnancy UAA: Urological Association of Asia; NICE: National Institute of Health and Care Excellence; EAU: European Association of Urology; AUA: American Urologic Association

Issue	UAA [30]	NICE [8]	EAU [7]	AUA [25]	Commentary
Choice of imaging modality	1^st^ line: Ultrasound 2^nd^ line: MRI Reserve low-dose CT as a last-line option	Offer Ultrasound instead of CT	1^st^ line: Ultrasound 2^nd^ line: 1.5T MRI 3^rd^ line: CT-scan (0.5mGy)	Radiation dose up to 50mGy allowable for imaging (or treatment) in pregnancy	CT scan at low dose (<0.5mGy) likely has an important role to clarify equivocal cases and prevent unnecessary invasive intervention such as ureteroscopy.
Choice of renal drainage procedure	Nephrostomy or Ureteric stenting	-	Nephrostomy or Ureteric stenting equally effective	Nephrostomy or Ureteric stenting equally effective	Percutaneous nephrostomy should probably be favoured over retrograde ureteric stenting as it seems better tolerated and can be carried out under local anaesthesia. In addition, it is easily performed solely under ultrasound guidance and in lateral position, thereby minimizing radiation exposure and supine hypotension syndrome.
Frequency of tube changes	-	-	-	-	Tube changes every 4-6 weeks seem to be ideal. Low threshold to increase frequency up to 3-weekly if necessary.
Ureteroscopy in pregnancy	Preferred treatment if conservative management fails	-	Can be considered by experienced urologist in specialist centre preferably in second trimester, with access to neonatal and obstetric service	Can be offered by experienced urologists in institutions with obstetric and neonatal support	Careful risk-benefit decision in multi-disciplinary setting is important
PCNL in pregnancy	Should be generally avoided	-	Individual decision, experienced centres	-	PCNL in pregnancy is probably only for exceptional cases when necessary.

An interesting, though uncommon, dilemma involves a management modality for patients who become pregnant while awaiting definitive stone or PUJO surgery and who already have a ureteric stent or nephrostomy in situ with ongoing significant tube irritation. Currently, there are no specific guidelines for this scenario, though it can be argued that there may be a role for counselling women of childbearing age who have drainage tubes in place to consider avoiding pregnancy until after definitive treatment. Alternatively, urologists can expedite definitive surgery in patients with tubes who are likely to conceive in the interim.

Challenges with pain management also introduce another dimension of complexity to the management of symptomatic hydronephrosis, especially in the third trimester. Opiates could cross the placenta and lead to neonatal respiratory depression and withdrawal symptoms, while NSAIDs have a risk of premature closure of the ductus arteriosus. For stent-related pain, Tamsulosin and Hyoscine have limited evidence base in pregnancy, while anticholinergics such as oxybutynin have a high side effect profile. Ultimately, there remains a limited choice of safe analgesia options in pregnancy, further worsening the quagmire.

## Conclusions

The management of symptomatic hydronephrosis in pregnancy is complex, especially in patients with ongoing symptoms, those who struggle to tolerate drainage tubes, and/or those prone to early encrustation. Urologists and other specialists often face the need to make important treatment decisions with a potential impact on both the mother and the baby. There are challenges related to diagnostic evaluation, pain control, and choice/timing of definitive treatment options. The risk of foetal compromise, coupled with limited evidence from the literature, further complicates the situation. There is probably a need for large collaborative studies to pool data and aid global guideline development for this relatively uncommon but important condition.
